# Association Between Disruption of Blood Pressure Circadian Rhythm and Prognosis After Acute Anterior Myocardial Infarction

**DOI:** 10.1002/clc.70377

**Published:** 2026-08-03

**Authors:** Yangge Shao, Yajuan Yang, Chao Wang, Zhiming Yu, Ruxing Wang

**Affiliations:** ^1^ Department of Cardiology, the Affiliated Wuxi People's Hospital of Nanjing Medical University, Wuxi People's Hospital, Wuxi Medical Center Nanjing Medical University Wuxi China

**Keywords:** anterior STEMI, blood pressure, circadian rhythm, MACE, PCI

## Abstract

**Objective:**

To investigate the characteristics of blood pressure (BP) circadian rhythm disruption and its relationship with prognosis in patients with acute anterior ST‐elevation myocardial infarction (STEMI) after emergency percutaneous coronary intervention (PCI).

**Methods:**

A total of 330 patients with anterior STEMI who underwent emergency PCI were enrolled in this study (MI group). Additionally, 131 physical examination patients were selected as the control group. Based on 24‐h ambulatory blood pressure monitoring (ABPM) results, BP rhythm in both groups was classified into Extreme dipper, Dipper, Non‐dipper, and Reverse dipper. The characteristics of BP rhythm were compared between the two groups. Patients with anterior MI were followed up for 1 year postoperatively. The occurrence of major adverse cardiovascular events (MACE) was compared among subgroups with different BP rhythms.

**Results:**

The proportion of dipper BP in the study group (11.21%) was significantly lower than that of the control group (63.36%) (*p* = 0.001). The proportions of non‐dipper (43.03%) and reverse dipper (40%) BP in the MI group were higher than the control group (22.90% and 7.63%, respectively; all *p* < 0.001). Multivariate Cox regression analysis, using dipper BP as the reference, showed that reverse dipper BP was independently associated with MACE (HR = 6.417, *p* = 0.002). The cumulative risk of the primary endpoint event (Log Rank *p* = 0.021) was significantly higher in the reverse dipper group compared to the dipper group.

**Conclusion:**

Patients with anterior STEMI are still under the burden of disrupted BP circadian rhythm and reduced dipper BP rhythm. The reverse dipper BP rhythm may serve as an independent factor of MACE.

## Graphical abstract

This study retrospectively analyzed the characteristics of blood pressure rhythm after emergency PCI for patients with anterior STEMI, discovering the burden of disrupted BP circadian rhythm. The reverse dipper BP rhythm may serve as an independent factor of MACE. This provides new insights into the intervention of blood pressure rhythm after myocardial infarction.

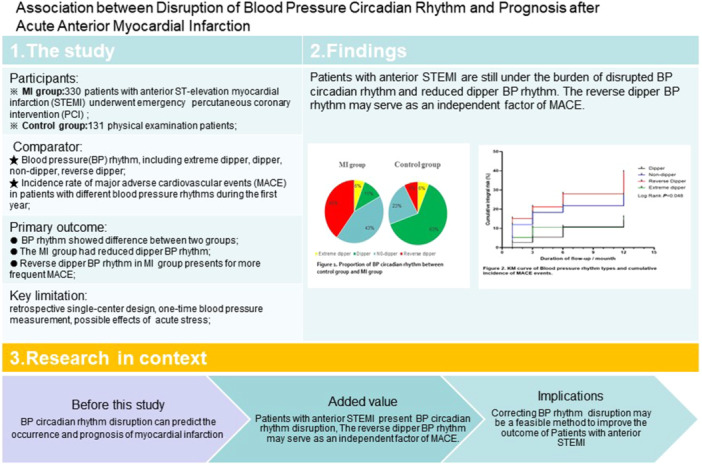

## 1 Introduction

Human blood pressure (BP) exhibits diurnal fluctuations over a 24‐h cycle, known as the BP circadian rhythm, characterized by a morning rise and a nocturnal fall [1]. In normal individuals, BP rhythm follows a “two‐peak, one‐valley” dipper pattern, peaking during waking daytime hours and falling during nocturnal sleep. A physiological nocturnal BP dip of approximately 10%–20% is termed “Dipper”. A dip less than 10% is termed “Non‐Dipper”, a dip exceeding 20% is termed “Extreme Dipper”, and a rise in nocturnal BP is termed “Reverse Dipper” [2, 3]. The Dipper pattern is generally considered the normal BP circadian rhythm, while Extreme Dipper, Non‐Dipper, and Reverse Dipper patterns represent pathological disruptions of the BP circadian rhythm. Disruption of the BP circadian rhythm is closely associated with the occurrence and development of cardiovascular and cerebrovascular diseases [4, 5, 6].

Acute ST‐elevation myocardial infarction (STEMI) is a critical condition with poor prognosis, and its incidence continues to rise, posing a severe threat to human life and health [7]. Although survival rates for STEMI patients have improved over the past two decades through percutaneous coronary intervention (PCI) and dual antiplatelet therapy (DAPT), post‐infarction complications remain a significant cause of high mortality and disability rates [8]. Acute anterior myocardial infarction, caused by severe lesions in the left anterior descending artery, involves larger areas of myocardial necrosis compared to infarctions in other locations, leading to more severe impacts on cardiac function and systemic hemodynamics, higher acute and long‐term mortality, and poorer prognosis [9]. Disruption of BP circadian rhythm is closely linked to cardiovascular disease. Previous studies have shown that BP circadian rhythm disruption has predictive value for the occurrence and prognosis of myocardial infarction [1, 4]. However, there is a lack of literature reporting on the characteristics of BP rhythm after myocardial infarction and its association with prognosis.

This study primarily explores the characteristics of BP circadian rhythm after emergency PCI for acute anterior myocardial infarction and its relationship with major adverse cardiovascular events (MACE), aiming to provide a reference for BP intervention and prognosis improvement in post‐MI PCI patients.

## 2 Method

### 2.1 Study Population

This was a retrospective study. We retrospectively enrolled 330 consecutive patients with acute anterior STEMI admitted to Wuxi People's Hospital, affiliated to Nanjing Medical University, between December 2018 and December 2023 as the study group. Concurrently, 131 patients who underwent physical examination and completed 24‐h ABPM at the Physical Examination Center of the same hospital were selected as the control group. Included patients met the following criteria: (1) First diagnosis of acute anterior STEMI; (2) Underwent emergency PCI within 12 h of symptom onset or beyond 12 h due to clinical and/or electrocardiographic evidence of ongoing myocardial ischemia; (3) In case of multivessel disease on coronary angiography, only the culprit left anterior descending artery vessel was treated; (4) The patient survived beyond the initial circadian cycle following PCI; (5) Drugs affecting blood pressure are taken in the morning (6:00–10:00 am), including calcium antagonists, beta‐blockers, RAAS blockers, and mineralocorticoid receptor antagonists. Exclusion criteria: (1) History of myocardial infarction, chronic heart failure, congenital heart disease, cardiomyopathy, or severe valvular heart disease; (2) Incomplete clinical data; (3) Failure to complete follow‐up successfully.

Acute anterior STEMI was defined as: (1) Meeting the diagnostic criteria for acute STEMI, including ischemic chest pain, new or presumed new significant ECG changes (ST‐segment elevation and T‐wave changes) in two or more contiguous leads, and abnormal cardiac biomarkers (elevation and/or fall of cardiac troponin values, with at least one value above the 99th percentile upper reference limit) [10, 11, 12]; (2) ECG showing ST‐segment elevation in leads V1–V4; coronary angiography confirming left anterior descending artery lesion; transthoracic echocardiography showing segmental wall motion abnormality in the anterior wall.

### 2.2 Clinical Data Acquisition

The following clinical parameters were collected via the electronic medical record system and imaging system after enrollment: (1) Basic information: gender, age, body mass index (BMI), diabetes, hypertension, dyslipidemia, smoking history, and family history of cardiovascular disease. (2) Results of initial blood laboratory tests, including: white blood cell count (WBC), neutrophil percentage (N%), C‐reactive protein (CRP), total cholesterol (TC), triglycerides (TG), low‐density lipoprotein cholesterol (LDL‐C), high‐density lipoprotein cholesterol (HDL‐C), estimated glomerular filtration rate (eGFR), cardiac troponin I (cTnI), and N‐terminal pro‐B‐type natriuretic peptide (NT‐proBNP). Peak biomarker values (including creatine kinase [CK] and CK‐MB) were obtained from repeated blood tests during hospitalization. (3) Initial transthoracic echocardiography data, including left ventricular ejection fraction (LVEF), left ventricular end‐diastolic diameter (LVEDD), left atrial diameter (LAD), etc. (4) Coronary angiography parameters: number of diseased vessels, Gensini score, TIMI risk score for STEMI [12], number of stents implanted, post‐PCI TIMI flow, and symptom‐to‐reperfusion time. Coronary angiography was analyzed by two independent interventional cardiologists. (5) BP rhythm classification: For the control group, classification was based on the 24‐h ABPM results.

For MI patients, classification was based on the first 24‐h ABPM measurement after admission (first daytime period 06:00–22:00 to next morning 06:00). Daytime was defined as 06:00–22:00, and nighttime as 22:00–06:00. BP rhythm types were defined based on the nocturnal SBP dipping percentage: Dipper (> 10% to 20%), Extreme dipper (> 20%), Non‐dipper (0% to 10%), Reverse dipper (< 0%) [13]. (6) Follow‐up endpoint and definition: All patients were followed up from the end of the procedure until 12 months post‐PCI via medical record review or telephone consultation to monitor MACE occurrence at 1, 3, 6, 9, and 12 months. MACE was defined as a composite of all‐cause death, recurrent myocardial infarction, unplanned repeat revascularization, and hospitalization for heart failure [14]. If a subject experienced multiple MACE events, only the first event was recorded.

### 2.3 Statistical Analysis

Statistical analysis was performed using SPSS software version 26.0. Normally distributed continuous data were expressed as mean ± standard deviation (mean ± SD) and compared between two groups using the independent samples *t*‐test. Comparisons among multiple groups were performed using analysis of variance (ANOVA). Non‐normally distributed data were expressed as median and interquartile range (IQR) and compared between two groups using the Mann‐Whitney *U* test. Comparisons among multiple groups were performed using the Kruskal‐Wallis test. Categorical data were described as frequency and percentage, and compared using the *χ* ^2^ test. Univariate Cox proportional hazards regression analysis was used to screen potential risk factors for endpoint events. Multivariate Cox regression analysis was used to investigate the relationship between BP circadian rhythm and endpoint events. Kaplan‐Meier curves were used to display the cumulative incidence of endpoint events for different BP circadian rhythms. A *p* value < 0.05 was considered statistically significant.

## 3 Results

### 3.1 Baseline Characteristics of the Study Patients

A total of 461 patients were included: 131 in the control group and 330 in the MI group. Compared to controls, the MI group had a significantly higher proportion of males and smokers, higher BMI, and higher prevalence of hypertension, diabetes, and dyslipidemia (all *p* < 0.01). There was no significant difference in the family history of cardiovascular disease between groups. Regarding blood tests and CRP, the MI group had significantly higher WBC count, neutrophil percentage, and CRP values than controls (all *p* < 0.01). Regarding lipids, TC and LDL‐C were significantly higher in the MI group (*p* < 0.01), while TG and HDL‐C showed no significant difference (*p* > 0.05). eGFR showed no significant difference. Echocardiography showed significantly larger LAD and LVEDD and significantly lower LVEF in the MI group compared to controls (all *p* < 0.01). as revealed in Table 1.

**Table 1:** Baseline clinical characteristics.

Variables	Control group (*n* = 131)	MI group (*n* = 330)	*t/χ* ^2^ */Z* Value	*p*‐Value
Age (years)	58.73 ± 7.52	57.95 ± 13.18	0.797	0.426
Male sex	66 (50.38%)	289 (87.56%)	73.266	< 0.001*
BMI (kg/m^2^)	24.05 ± 2.95	25.19 ± 3.35	−3.597	< 0.001*
Cardiovascular risk factors				
Current smoking	33 (25.19%)	190 (57.58%)	39.381	< 0.001*
Family history of IHD	4 (3.05%)	18 (5.45%)	1.190	0.275
Hypertension	30 (22.90%)	181 (54.85%)	38.561	< 0.001*
Diabetes	15 (11.45%)	84(25.45%)	10.906	0.001*
Dyslipidemia	38 (29.01%)	119(36.06%)	2.077	0.015*
Laboratory data				
WBCs (× 109/L)	7.25 ± 2.73	11.38 ± 3.59	−13.333	< 0.001*
N%	68.32 ± 6.24	77.21 ± 8.33	−12.479	< 0.001*
CRP (mg/L)	1.8 (0.6,5.6)	4.8 (1.2,12.1)	−4.018	< 0.001*
TC (mmol/L)	4.56 ± 1.17	4.97 ± 1.53	−3.095	0002*
TG (mmol/L)	1.43 (0.72,1.83)	1.50 (1.08,2.10)	−0.685	0.494
LDL‐C (mmol/L)	2.48 ± 0.72	3.08 ± 1.02	−7.116	< 0.001*
HDL‐C (mmol/L)	1.12 ± 0.26	1.08 ± 0.31	1.408	0.160
eGFE (mL/(min.1.73 m^2^))	96.52 ± 16.52	91.45 ± 21.78	2.702	0.170
Echocardiographic data				
LAD(mm)	33.21 ± 4.22	36.01 ± 4.57	−6.273	< 0.001*
LVEDD(mm)	41.52 ± 4.61	47.91 ± 4.84	−13.232	< 0.001*
LVEF(%)	55.22 ± 5.46	49.82 ± 7.28	8.667	< 0.001*
24 h ABPM averages				
SBP (mmHg)	127.68 ± 7.66	115.99 ± 19.65	9.190	< 0.001*
DBP (mmHg)	78.01 ± 6.42	73.91 ± 11.65	4.812	< 0.001*
Blood pressure rhythm types				
Dipper	83 (63.36%)	37(11.21%)	132.435	< 0.001*
Extreme dipper	8 (6.11%)	19(5.76%)	0.021	0.885
Non‐dipper	30 (22.90%)	142(43.03%)	16.245	< 0.001*
Reverse Dipper	10 (7.63%)	132(40%)	46.089	< 0.001*

Abbreviations: ABPM, Ambulatory blood pressure; BMI, Body mass index; DBP, Diastolic blood pressure; eGFR, Estimated glomerular filtration rate; HDL‐C, high‐density lipoprotein cholesterol; IHD, ischemic heart disease; LAD, Left atrial diameter; LDL‐C, low‐density lipoprotein cholesterol; LVEDD, left ventricular end‐diastolic diameter; LVEF, left ventricular ejection fraction; *N*%, Neutrophil percentage; SBP, systolic blood pressure; TC, Total cholesterol; TG, Triglycerides; WBC, White blood cell count.

* *p* < 0.05.

### 3.2 Characteristics of BP Circadian Rhythm After MI

The control group had significantly higher SBP than the MI group (both *p* < 0.01). The Dipper pattern predominated in controls (63.36%), while Non‐Dipper (43.03%) and Reverse Dipper (40%) patterns predominated in the MI group. The proportion of Dipper pattern was significantly lower in the MI group (11.21%) than in controls (63.36%) (*p* < 0.01), while the proportions of Non‐Dipper (43.03% vs 22.90%) and Reverse Dipper (40.00% vs 7.63%) patterns were significantly higher in the MI group (both *p* < 0.01). There was no significant difference in the Extreme Dipper proportion (*p* > 0.05). as revealed in Table 1.

### 3.3 Characteristics Across BP Rhythm Groups Within the MI Group

As shown in Table 2, there were no significant differences in age, gender distribution, or BMI among the Dipper, Extreme Dipper, Non‐Dipper, and Reverse Dipper subgroups within the MI group (all *p* > 0.05). Among cardiovascular risk factors, the proportions of hypertension, dyslipidemia, family history of IHD, and smoking showed no significant differences between groups (all *p* > 0.05), but the proportion of diabetes differed (*p* < 0.05), being highest in the Non‐Dipper group (32.39%). Regarding laboratory indices, only CRP differed significantly between groups (*p* < 0.01); WBC count, neutrophil percentage, lipids, eGFR, peak CK, peak CK‐MB, and NT‐proBNP showed no significant differences (all *p* > 0.05). Echocardiographic indices (LAD, LVEDD, LVEF) also showed no significant differences. Regarding MI treatment, there were no significant differences in symptom‐to‐reperfusion time, number of diseased vessels, Gensini score, TIMI risk score, number of stents implanted, or proportion achieving post‐PCI TIMI 3 flow (all *p* > 0.05). The proportion using RAAS blockers differed significantly (*p* < 0.01), being highest in the Extreme Dipper group (89.47%). Proportions using β‐blockers or MRAs showed no differences. MACE incidence differed significantly (*p* < 0.01), being lowest in the Dipper group (13.51%) and highest in the Reverse Dipper group (39.39%).

**Table 2:** Characteristics of different BP rhythms after MI.

Variables	Dipper (*n* = 37)	Extreme dipper (*n* = 19)	Non‐dipper (*n* = 142)	Reverse dipper (*n* = 132)	*H/F/χ* ^2^	*p*‐value
Age (years)	60.38 ± 13.13	54.84 ± 13.20	56.50 ± 13.54	59.30 ± 12.71	1.83	0.14
Male sex	29 (78.38%)	16 (84.21%)	124 (87.32%)	120 (90.91%)	1.90	0.54
BMI (kg/m^2^)	24.91 ± 3.25	24.87 ± 3.29	25.19 ± 3.13	25.31 ± 3.43	0.20	0.90
Cardiovascular risk factors						
Current smoking	19 (51.35%)	12 (63.16%)	83 (58.45%)	76 (57.58%)	0.87	0.83
Family history of IHD	2 (5.41%)	0 (0%)	9 (6.34%)	7 (5.30%)	1.32	0.73
Hypertension	20 (54.05%)	12 (63.16%)	69 (48.59%)	80 (60.61%)	3.64	0.30
Diabetes	4 (10.81%)	3 (15.79%)	46 (32.39%)	31 (23.48%)	8.99	0.03*
Dyslipidemia	9 (24.32%)	4 (21.05%)	58 (40.85)	48 (36.36%)	5.48	0.14
Laboratory data						
WBCs (× 109/L)	11.41 ± 3.01	10.24 ± 3.42	11.87 ± 4.03	11.02 ± 3.09	2.12	0.10
N%	72.80 (70.35, 81.40)	77.10 (66.40, 83.30)	79.20 (72.08, 83.13)	79.3 (72.08, 83.78)	5.57	0.14
CRP (mg/L)	8.40 (2.20, 19.55)	6.8 (0.90, 13.20)	5.0 (1.40, 13.90)	3.15 (1.50, 8.38)	11.05	0.01*
TC (mmol/L)	4.88 ± 1.62	4.85 ± 1.24	4.49 ± 1.68	5.12 ± 1.20	0.31	0.96
TG (mmol/L)	1.19 (0.94, 1.77)	1.34 (1.20, 1.96)	1.50 (1.11, 2.07)	1.55 (1.12, 2.25)	1.02	0.80
LDL‐C (mmol/L)	3.09 ± 1.14	2.96 ± 1.07	3.07 ± 0.99	3.11 ± 1.01	0.14	0.94
HDL‐C (mmol/L)	0.99 ± 0.20	1.04 ± 0.16	1.08 ± 0.28	1.10 ± 0.31	5.31	0.15
eGFE (ml/ (min.1.73 m^2^))	96.60 (84.65, 106.00)	99.70 (80.10, 107.40)	96.0 (81.20, 107.15)	93.9 (83.85, 104.4)	3.42	0.33
Peak CK (ng/mL)	3119 (1402, 5815)	4613 (1472, 5607)	3234 (1472, 5292)	3470.5 (1201, 5119)	0.31	0.96
Peak CK‐MB (ng/mL)	253 (166, 549)	275 (168, 446)	253.6 (151, 406)	270 (117, 428)	0.64	0.87
NT‐proBNP (ng/L)	2090 (654.5, 6800)	1230 (689, 3470)	1520 (493, 5300)	1200 (360, 4440)	4.42	0.22
LAD (mm)	35.89 ± 4.71	35.21 ± 2.99	35.79 ± 4.82	36.39 ± 4.45	0.60	0.62
LVEDD (mm)	47.86 ± 4.28	46.58 ± 3.42	47.64 ± 4.81	48.41 ± 5.86	1.07	0.36
LVEF (%)	49.73 ± 7.80	48.21 ± 6.92	49.91 ± 7.01	49.98 ± 7.51	0.34	0.80
24 h ABPM averages						
SBP (mmHg)	109.05 ± 15.43	95.96 ± 16.65	112.01 ± 17.88	125.04 ± 18.79	23.27	< 0.01*
DBP (mmHg)	69.97 ± 7.81	63.67 ± 10.94	72.20 ± 11.31	78.29 ± 11.35	15.22	< 0.01*
Recanalization time (h)	5 (2.75, 8.00)	4.5 (3.00, 8.00)	4 (2.00, 6.25)	3.5 (2.00, 6.00)	5.08	0.17
Number of diseased vessels	1.843 ± 0.87	1.533 ± 0.60	1.553 ± 0.69	1.583 ± 0.69	2.28	0.52
Number of Stents Implanted	1.16 ± 0.37	1.05 ± 0.40	1.16 ± 0.48	1.22 ± 0.53	2.49	0.48
Post‐PCI TIMI 3 flow	33 (89.19%)	17 (89.47%)	131 (92.25%)	120 (90.91%)	3.57	0.73
Gensini score	80 (48.00, 90.00)	80 (48.00, 90.00)	80 (58.00, 84.00)	80 (52, 84.75)	0.95	0.82
TIMI risk score	4.0 (2, 5)	4.5 (3, 8)	3.5 (2, 5)	3 (2, 5)	0.51	0.92
β‐blocker	25 (67.57%)	15 (78.95%)	111 (78.17%)	105 (79.55%)	2.47	0.48
RAAS ‐blocker	21 (56.76%)	17 (89.47%)	55 (38.73%)	77 (58.33%)	9.27	0.05*
MRA	3 (8.11%)	1 (5.26%)	17 (11.97%)	13 (9.85%)	1.54	0.67
MACE	5 (13.51%)	3 (15.79%)	39 (27.46%)	52 (39.39%)	12.60	< 0.01*

Abbreviations: ABPM, Ambulatory blood pressure; BMI, Body mass index; CK, Creatine kinase; CK‐MB, creatine kinase‐myocardial band; DBP, Diastolic blood pressure; eGFR, Estimated glomerular filtration rate; HDL‐C, high density lipoprotein cholesterol; IHD, ischemic heart disease; LAD, Left atrial diameter; LDL‐C, low density lipoprotein cholesterol; LVEDD, left ventricular end‐diastolic diameter; LVEF, left ventricular ejection fraction; MRA, Mineralocorticoid antagonist; NT‐proBNP, N‐terminal pro‐B‐type natriuretic peptide; N%, Neutrophil percentage; RAAS blocker, renin‐angiotensin‐aldosterone system; SBP, systolic blood pressure; TC, Total cholesterol; TG, Triglycerides; WBC, White blood cell count.

* *p* < 0.05.

### 3.4 Relationship Between Clinical Prognosis and Risk Factors

**Figure 1:**
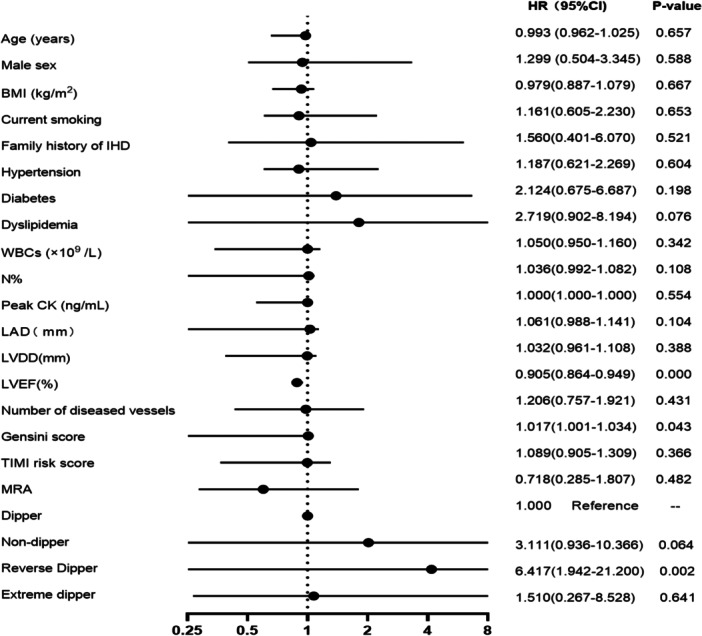
Multivariate COX regression analysis of the MACE.

Table 3 shows the results of univariate Cox regression analysis for MACE. Per unit increase: WBC count (HR 1.091, *p* = 0.009), Neutrophil percentage (HR 1.065, *p* < 0.01), Peak CK (HR 1.0, *p* < 0.01), LAD (HR 1.108, *p* < 0.01), LVEDD (HR 1.105, *p* < 0.01), Number of diseased vessels (HR 1.593, *p* < 0.01), Gensini score (HR 1.025, *p* < 0.01), TIMI risk score (HR 1.137, *p* < 0.05). Per unit decrease: LVEF (HR 0.888, *p* < 0.01). MRA use (HR 0.465, *p* = 0.035) and Reverse Dipper rhythm (HR 4.160, *p* < 0.01) were identified as potential risk factors for endpoint events. Potential risk factors from univariate analysis plus clinically recognized high‐risk factors (age, sex, hypertension, diabetes, dyslipidemia, smoking, BMI, family history of CVD) [15] were included in multivariate Cox regression analysis. Results (Figure 1) showed that increased LVEF (HR 0.905, *p* < 0.01 per 1% increase), increased Gensini score (HR 1.017, *p* = 0.043 per 1 score increase), and Reverse Dipper rhythm (using Dipper as reference, HR 6.417, *p* = 0.002) were independent risk factors for MACE.

**Table 3:** Univariate COX regression analysis of MACE.

Variables	B	SE	Wald	*p*‐value	HR	95% CI
Age (years)	0.009	0.009	0.918	0.338	1.009	0.991–1.027
Male sex	0.081	0.342	0.056	0.813	1.084	0.555–2.119
BMI (kg/m^2^)	−0.031	0.037	0.737	0.391	0.969	0.902–1.041
Cardiovascular risk factors						
Current smoking	0.059	0.243	0.059	0.808	1.061	0.659–1.707
Family history of IHD	0.427	0.580	0.541	0.462	1.532	0.491–4.777
Hypertension	0.017	0.241	0.005	0.942	1.018	0.634–1.633
Diabetes	0.015	0.276	0.003	0.956	1.015	0.591–1.745
Dyslipidemia	0.235	0.254	0.855	0.355	1.265	0.769–2.082
Laboratory data						
WBCs (×10^9^/L)	0.087	0.033	6.835	0.009*	1.091	1.022–1.164
*N*%	0.063	0.017	14.196	< 0.01*	1.065	1.031–1.100
CRP (mg/L)	0.001	0.006	0.013	0.908	1.001	0.989–1.012
TC (mmol/L)	−0.007	0.016	0.176	0.675	0.993	0.963–1.025
TG (mmol/L)	0.042	1.00	0.179	0.672	1.043	0.858–1.269
LDL‐C (mmol/L)	0.025	0.118	0.045	0.833	1.025	0.814–1.292
HDL‐C (mmol/L)	−0.004	0.305	0.000	0.990	0.996	0.548–1.812
eGFE (ml/min*1.73 m^2^)	−0.008	0.005	2.306	0.129	0.992	0.981–1.002
Peak CK (ng/mL)	0.000	0.000	6.422	0.011*	1.00	1.000–1.000
Peak CK‐MB (ng/mL)	0.001	0.000	3.815	0.051	1.001	1.00–1.001
NT‐proBNP (ng/L)	0.000	0.000	1.141	0.285	1.00	1.00–1.000
LAD (mm)	0.103	0.027	14.087	< 0.01*	1.108	1.050–1.170
LVEDD (mm)	0.10	0.026	14.154	< 0.01*	1.105	1.049–1.164
LVEF (%)	−0.119	0.020	36.077	< 0.01*	0.888	0.854–0.923
Recanalization time (h)	−0.033	0.028	1.334	0.248	0.968	0.915–1.023
Number of diseased vessels	0.465	0.167	7.771	0.005*	1.593	1.148–2.209
Number of Stents Implanted	0.078	0.245	0.103	0.749	1.081	0.670–1.747
Post‐PCI TIMI 3 flow	−0.285	0.414	0.473	0.492	0.752	0.334–1.693
Gensini score	0.024	0.006	18.109	< 0.01*	1.025	1.013–1.036
TIMI risk score	0.128	0.056	5.218	0.022*	1.137	1.018–1.269
β‐blocker	−0.227	0.282	0.649	0.421	0.797	0.459–1.384
RAAS ‐blocker	−0.294	0.242	1.473	0.225	0.746	0.464–1.198
MRA	−0.766	0.363	4.447	0.035*	0.465	0.228–0.947
24 h ABPM averages						
SBP (mmHg)	−0.001	0.004	0.110	0.740	0.999	0.990–1.007
DBP (mmHg)	0.001	0.008	0.012	0.911	1.001	0.985–1.017
Blood pressure rhythm types						
Dipper (reference)						
Non‐dipper	0.885	0.516	2.939	0.086	2.423	0.881–6.667
Reverse Dipper	1.426	0.513	7.727	0.005*	4.160	1.523–11.366
Extreme dipper	0.182	0.792	0.053	0.818	1.200	0.254–5.665

Abbreviations: ABPM, Ambulatory blood pressure; BMI, Body mass index; CK, Creatine kinase; CK‐MB, creatine kinase‐myocardial band; DBP, Diastolic blood pressure; eGFR, Estimated glomerular filtration rate; HDL‐C, high density lipoprotein cholesterol; IHD, ischemic heart disease; LAD, Left atrial diameter; LDL‐C, low density lipoprotein cholesterol; LVEDD, left ventricular end‐diastolic diameter; LVEF, left ventricular ejection fraction; MRA, Mineralocorticoid antagonist; NT‐proBNP, N‐terminal pro‐B‐type natriuretic peptide; *N*%, Neutrophil percentage; RAAS blocker, renin‐angiotensin‐aldosterone system; SBP, systolic blood pressure; TC, Total cholesterol; TG, Triglycerides; WBC, White blood cell count.

* *p* < 0.05.

### 3.5 Relationship Between BP Rhythm and Endpoint Events

**Figure 2:**
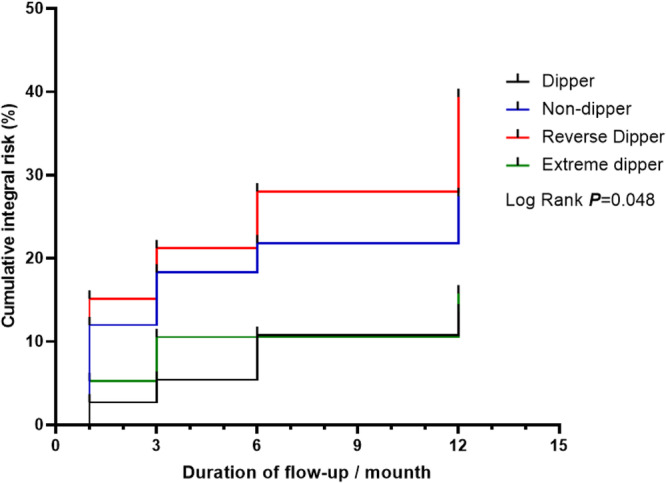
KM curve of Blood pressure rhythm types and cumulative incidence of MACE events.

After 12 months of follow‐up, 99 MACE events occurred cumulatively: 8 all‐cause deaths, 2 recurrent MIs, 56 unplanned repeat revascularizations, and 27 hospitalizations for heart failure. MACE incidence rates were: Dipper 13.51%, Extreme Dipper 15.79%, Non‐Dipper 27.46%, Reverse Dipper 39.39%. Compared to the Dipper group (13.51%), MACE incidence in the Extreme Dipper (15.79%) and Non‐Dipper (27.46%) groups showed no significant difference (*p* > 0.05), but was significantly higher in the Reverse Dipper group (39.39%, *p* < 0.01). Figure 2 shows the Kaplan‐Meier cumulative risk curves for different BP rhythms. The cumulative risk of MACE differed significantly across BP rhythm groups (Log Rank *p* = 0.048). The Dipper group had the lowest cumulative risk, the Reverse Dipper group the highest, and the cumulative risk in the Reverse Dipper group was significantly higher than in the Dipper group (Log Rank *p* = 0.021).

## 4 Discussion

In healthy individuals, blood pressure is regulated by clock genes such as *Bmal1*, *Per1/2*, *Clock*, and *Cry*, exhibiting a distinct circadian rhythm with peaks during the awake daytime and troughs during nighttime sleep [16]. Based on the nocturnal SBP dipping percentage, BP circadian rhythm is classified into four types: Dipper, Extreme dipper, Non‐dipper, and Reverse dipper. According to literature, slightly more than half of hypertensive patients are dippers at baseline, about one‐quarter to one‐third are non‐dippers, and less than 10% are reverse dippers [17, 18], consistent with our findings in the control group. Factors such as age, shift work, circadian misalignment, social jet lag, genetic variants/mutations, and cardiovascular/cerebrovascular diseases can influence BP rhythm [2, 19].

Acute Anterior STEMI causes extensive myocardial necrosis, leading to overactivation of the neuroendocrine system, predisposing patients to cardiac dysfunction and systemic hemodynamic disturbances [20]. Our study on BP rhythm characteristics after emergency PCI for acute anterior MI revealed that BP rhythm disruption occurs as early as the first day post‐PCI. While dipper BP was predominant in controls, non‐dipper and reverse dipper patterns predominated in the MI group. Compared to controls, the MI group had a significantly lower proportion of dippers and significantly higher proportions of non‐dippers and reverse dippers. No significant difference was found in the proportion of extreme dippers.

There are clear differences between the control group and the myocardial infarction group in terms of sex distribution, body mass index, and cardiovascular risk factors. These differences are not the main factors influencing blood pressure rhythms, and these comparisons are descriptive rather than intended to show a causal relationship. Numerous studies have confirmed that the blood pressure rhythm is mainly the combined result of both endogenous circadian mechanisms, as well as 24‐h environmental and behavioral variations, such as the sleep/wake, rest/activity, fasting/eating, dark/light, and postural, mental stress, and environmental cycles [21]. For patients with acute myocardial infarction, according to the Guideline‐directed medical therapy, they usually need to take drugs such as beta‐blockers, RAAS blockers, and mineralocorticoid receptor antagonists to improve prognosis. The effects of these drugs and their dosing times on nocturnal blood pressure and blood pressure rhythm are still controversial. Ye discovered that olmesartan and amlodipine administered before bedtime can better control nocturnal blood pressure and improve the circadian rhythm [22]. However, currently, most experts do not recommend taking antihypertensive drugs before bedtime, mainly because most outcome trials use morning administration, and there is no clear and strong evidence indicating that bedtime administration has additional outcome benefits. This study uses morning administration, and the drugs taken are all long‐acting preparations. In most individuals, they are likely to remain above the treatment threshold within 24 h. Therefore, theoretically, the type and time of administration should not have any impact on the efficacy. The anterior STEMI BP rhythm disruption likely results from multiple interacting factors. Firstly, anterior STEMI causes large‐scale ischemic necrosis of the left ventricular anterior wall, damaging mechanoreceptors and leading to decreased baroreflex sensitivity. This subsequently triggers sympathetic overactivation and inhibition of vagal tone [20]. This autonomic imbalance causes abnormal nocturnal norepinephrine release, phase shifts in cortisol secretion, and loss of diurnal differences in nitric oxide bioavailability [23, 24]. Secondly, inflammatory mediators released during MI inhibit the expression of the CLOCK/BMAL1 complex in cardiomyocytes via the IL‐6/STAT3 signaling pathway, leading to the loss of circadian secretion rhythm of angiotensinogen (AGT) [25, 26, 27, 28]. Additionally, MI disrupts sleep patterns [29] and “inhibits” the rhythm‐regulating function of the suprachiasmatic nucleus (SCN) [26, 30], causing dysfunction of clock gene BMAL1 [25]and vascular smooth muscle cell PRDM16 [31, 32]. These multifactorial mechanisms collectively contribute to BP rhythm disruption post‐MI.

In this study, we further analyzed the characteristics of patients with different BP rhythms post‐MI. Apart from differences in diabetes prevalence, no statistically significant differences were found among the BP rhythm groups regarding age, gender, BMI, or common cardiovascular risk factors. This might be because diabetes itself, through a series of pathophysiological processes including inflammation, oxidative stress, and insulin resistance, can exacerbate BP rhythm disruption [33, 34]. As far as the laboratory indicators are concerned, CRP, reflecting inflammation, showed a difference between groups, with dipper patients seemingly having higher levels, which contradicts previous findings [35], which suggests that post‐MI BP rhythm is complex, influenced by numerous factors beyond inflammation alone.

Regarding MI severity assessment, PCI procedure, and medication regimens, there were no significant differences among the BP rhythm groups. However, outcome data clearly showed the lowest MACE incidence in the dipper group and the highest in the reverse dipper group, indicating that BP rhythm disruption likely impacts MACE occurrence in MI patients. Univariate Cox regression analysis identified WBC count, neutrophil percentage, peak CK, LAD, LVEDD, LVEF, number of diseased vessels, Gensini score, TIMI risk score, MRA use, and reverse dipper rhythm as potential risk factors for MACE post‐MI. Incorporating these potential risk factors along with demographic characteristics and common risk factors into a multivariate Cox regression analysis revealed that LVEF, Gensini score, and reverse dipper BP rhythm were independently associated with MACE.

The association of LVEF and Gensini score with post‐MI MACE is well‐established by previous studies [9, 36, 37]. The JAMP study [38] showed that disrupted BP circadian rhythm (reverse dipping) was significantly associated with higher overall cardiovascular risk, compared to normal circadian rhythm (dipping). This aligns with our findings. Our study demonstrated that reverse dipper BP increased the risk of MACE by 6.42‐fold compared to dipper BP (HR = 6.42, 95%CI 1.94–21.20). The Kaplan‐Meier cumulative risk curve also showed higher cumulative risk for endpoint events in the reverse dipper group. We therefore conclude that in acute anterior MI, the reverse dipper BP rhythm is independently associated with the occurrence of MACE events. Other types of disrupted BP rhythm (non‐dipper, extreme dipper) did not show independent association with MACE events, possibly because reverse dipping represents a more severe form of circadian disruption with greater detrimental cardiovascular impact.

Numerous studies indicate that reverse dipper hypertension is an independent risk factor for cardiovascular death. Potential mechanisms for poor prognosis in reverse dipper patients post‐MI are as follows: Firstly, elevated nocturnal BP keeps the cardiovascular system under constant high load, exacerbating endothelial damage and arterial stiffness [39]. Secondly, reverse dipping is associated with nocturnal autonomic dysfunction, characterized by sympathetic overactivation, impaired baroreflex sensitivity, sodium and water retention, and nocturnal volume overload [40, 41, 42]. Furthermore, nocturnal BP elevation can lead to excessive activation of inflammatory responses and disruption of clock gene expression, all of which can increase MACE incidence [26, 29], increasing MACE risk. Recent research by Ruan et al [43]demonstrated that ischemia‐reperfusion injury following acute anterior STEMI follows a circadian pattern. Administering treatments timed according to this rhythm (“chronotherapy”) can significantly improve patient outcomes. The circadian pattern of post‐MI ischemia‐reperfusion injury resembles the BP rhythm patterns we studied, and both are regulated by the clock gene BMAL1. Whether correcting disrupted BP circadian rhythm in post‐MI patients using antihypertensive or vasoactive drugs timed to the rhythm can improve prognosis warrants further exploration.

However, this study also has some limitations. As a single‐center retrospective study with a relatively small sample size, potential biases exist, necessitating confirmation by large‐scale randomized controlled trials (RCTs). Secondly, defining daytime and nighttime using fixed time intervals, although endorsed by relevant guidelines and validated by some researchers [44], may not reflect the true circadian patterns of individual patients, potentially introducing misclassification of BP rhythm types. Thirdly, Blood pressure circadian rhythm was assessed only during the first 24 h after myocardial infarction. This period is often affected by pain, stress, inflammation, and early post‐PCI instability. Therefore, the measured blood pressure pattern may reflect an acute response rather than the patient's usual circadian rhythm. Last but not least, the 24‐h blood pressure rhythm after PCI of acute myocardial infarction was identified as a research topic in this study, with the limitation of difficulties for repeated measurement.

## 5 Conclusion

According to this study, in spite of aggressive PCI therapies in time, patients with acute anterior wall myocardial infarction are still under the burden of disrupted BP circadian rhythm and reduced dipper BP rhythm. Furthermore, the reverse dipper BP rhythm may serve as an independent factor of MACE. This study could provide guidance for blood pressure management and prognosis improvement in patients with anterior wall myocardial infarction.

## Author Contributions

Yangge Shao wrote the main manuscript text; Yangge Shao, Yajuan Yang, and Chao Wang collected and analyzed the data; Zhiming Yu and Ruxing Wang put forward revision comments on the shortcomings of the article. All authors read and approved the final manuscript.

## Ethics Statement

The studies were conducted in accordance with the local legislation and institutional requirements. The participants provided their written informed consent to participate in this study.

## Conflicts of Interest

The authors declare no conflicts of interest.

## Data Availability

The data that support the findings of this study are available from the corresponding author upon reasonable request. The raw data supporting the conclusions of this article will be made available by the authors, without undue reservation.
